# TRIM21-regulated Annexin A2 plasma membrane trafficking facilitates osteosarcoma cell differentiation through the TFEB-mediated autophagy

**DOI:** 10.1038/s41419-020-03364-2

**Published:** 2021-01-06

**Authors:** Huan-Tian Zhang, Qingzhong Zeng, Baomeng Wu, Junlei Lu, Kui-Leung Tong, Jiebin Lin, Qiu-Yu Liu, Lipei Xu, Jie Yang, Xiaohui Liu, Wanting Liu, Yun-Fang Zhang, Qionghua Lian, Langxia Liu, Xuejuan Gao

**Affiliations:** 1 Key Laboratory of Functional Protein Research of Guangdong Higher Education Institutes and MOE Key Laboratory of Tumor Molecular Biology, Institute of Life and Health Engineering, Jinan University, Guangzhou, 510632 China; 2 Department of Bone and Joint Surgery, Institute of Orthopedic Diseases, The First Affiliated Hospital, Jinan University, Guangzhou, Guangdong 510630 China; 3 Department of Pathology, Henan Provincial People’s Hospital, People’s Hospital of Zhengzhou University, 7 Weiwu Road, Zhengzhou, 450003 China; 4 Center of Kidney Disease, Huadu District People’s Hospital, Southern Medical University, Guangzhou, Guangdong 510800 China

**Keywords:** Bone cancer, Oncogenes, Macroautophagy, Differentiation

## Abstract

Osteosarcoma (OS) is the most common primary malignant bone tumor in children and adolescents, which is characterized by dysfunctional autophagy and poor differentiation. Our recent studies have suggested that the tripartite motif containing-21 (TRIM21) plays a crucial role in regulating OS cell senescence and proliferation via interactions with several proteins. Yet, its implication in autophagy and differentiation in OS is largely unknown. In the present study, we first showed that TRIM21 could promote OS cell autophagy, as determined by the accumulation of LC3-II, and the degradation of cargo receptor p62. Further, we were able to identify that Annexin A2 (ANXA2), as a novel interacting partner of TRIM21, was critical for TIRM21-induced OS cell autophagy. Although TRIM21 had a negligible effect on the mRNA and protein expressions of ANXA2, we did find that TRIM21 facilitated the translocation of ANXA2 toward plasma membrane (PM) in OS cells through a manner relying on TRIM21-mediated cell autophagy. This functional link has been confirmed by observing a nice co-expression of TRIM21 and ANXA2 (at the PM) in the OS tissues. Mechanistically, we demonstrated that TRIM21, via facilitating the ANXA2 trafficking at the PM, enabled to release the transcription factor EB (TFEB, a master regulator of autophagy) from the ANXA2-TFEB complex, which in turn entered into the nucleus for the regulation of OS cell autophagy. In accord with previous findings that autophagy plays a critical role in the control of differentiation, we also demonstrated that autophagy inhibited OS cell differentiation, and that the TRIM21/ANXA2/TFEB axis is implicated in OS cell differentiation through the coordination with autophagy. Taken together, our results suggest that the TRIM21/ANXA2/TFEB axis is involved in OS cell autophagy and subsequent differentiation, indicating that targeting this signaling axis might lead to a new clue for OS treatment.

## Introduction

Osteosarcoma (OS) is the most common primary bone malignancy in children and adolescents^1^. Although the survival rate of patients with a localized tumor can reach 70% at 5 years, for those who developed pulmonary metastatic or relapsed, its survivorship is significantly reduced to only 20–30%^2,3^. This poor clinical outcome underscores the urgent need to clarify the mechanism of OS pathogenesis aiming to develop novel therapeutic strategies. Increasing evidence has suggested that more than 80% of OS are poorly differentiated and a better-differentiated state is associated with a better prognosis of the patients^4,5^. Thus, exploring the molecular mechanism underlying differentiation defects in OS is insightful for OS therapy^4,5^.

The tripartite motif proteins (TRIM) have increasingly been recognized to play a role in regulating autophagy and are implicated in innate immune responses^6^. Among them, TRIM21 assembles autophagy machinery through interacting with several key components of the autophagic machinery and acts as an autophagic receptor^7^; whereas, its implication in tumor autophagy has not been investigated. Recent reports have shown that TRIM21 participates in the regulation of immune cell subset differentiation^8,9^; however, its effect on tumor cell differentiation has not been uncovered. A series of studies have highlighted the role of TRIM21 in multiple cancers. For instance, TRIM21 has been demonstrated to be downregulated in hepatocellular carcinoma, breast cancer, and diffuse large B-cell lymphomas^10–13^, whereas other studies have shown that decreased expression of TRIM21 predicts better prognosis in pancreatic cancer patients^14^. We have recently demonstrated that TRIM21 plays a crucial role in regulating OS cell senescence and proliferation via interacting with several proteins, such as PRMT5^15,16^. However, it remains to be investigated whether TRIM21 plays a role in regulating OS autophagy and subsequent differentiation.

In this study, we first demonstrated that TRIM21 played a crucial role in regulating OS autophagy. Mechanistically, we found that TRIM21, via interacting with Annexin A2 (ANXA2), regulated its plasma membrane (PM) localization, which in turn, facilitated the nuclear translocation of transcription factor EB (TFEB). Altogether, we propose that the TRIM21/ANXA2/TFEB axis regulates OS cell differentiation via the induction of autophagy.

## Materials and methods

### Antibodies and reagents

The following antibodies were obtained from Cell Signaling Technology (USA): p62 (#23214), Beclin 1 (#3495), LAMP1 (#9091), HistonH3 (#4499), atp A1 (#96292), GFP (#2555), and LC3-II (#2775). Anti-TRIM21 (sc-25351) was from Santa Cruz Biotechnology (USA). Antibodies against RUNX2 (ab23981) and TFEB (ab220695) were from Abcam (USA). Antibodies against ANXA2 (11256-1-AP, 66035-1-lg), CD63 (25682-1-AP), β-actin (20536-1-AP), β-tubulin (10094-1-AP), and GAPDH (10494-1-AP) were from Proteintech (USA). Antibodies against LC3 (L7543), Flag (SAB4301135), and HA (H6908) were from Sigma (USA). Alexa Fluor 488- and 594-conjugated secondary antibodies (ZF-0511, ZF-0512, ZF-0516, ZF-0513) were from ZSGB-BIO (China). Chloroquine phosphate (CQ, PHR1258-1G), tetracycline (TET, #58346), 3-MA (M9281), MG132 (#474790), rapamycin (RAPA) (V900930-1mg), and DAPI (#10236276001) were from Sigma (USA).

### Cell lines and culture

Human OS U2-OS and Saos-2 cells (ATCC, USA) were maintained in McCoy’s 5A medium (Sigma, USA) containing 10% or 15% fetal bovine serum (FBS, PAN-Biotech, Germany) in a humidified atmosphere containing 5% CO_2_. MG63 cells (Institute of Life Science Chinese Academy of Sciences, China) were cultured in MEM (Gibco BRL, USA) and supported with 10% FBS. All these cells were authenticated by short tandem repeat (STR) as described previously^17^ and were mycoplasma-free tested by TransDetect® PCR Mycoplasma Detection Kit (TRANS, China).

### Small interfering RNA transfection

Sequences of the small interfering RNAs (siRNAs) used in this study were listed as follows: 5′-GCAGGAGUUGGCUGAGAAGTT-3′ (TRIM21), 5′-GGGUCUGUCAAAGCCUAUATT-3′ (ANXA2), 5′-CAGTTTGGCACAATCAATATT-3′ (Beclin 1), 5′-AGACGAAGGUUCAACAUCA-3′ (TFEB), and 5′-UUCUCCGAACGUGUCACGUTT-3′ (NC). OS cells were transfected with the siRNAs using Lipofectamine™2000 (Invitrogen, CA, USA) according to the manufacturer’s instructions.

### Establishment of OS stable cells

The U2-OS cells stably expressing H125-TRIM21 or H125-V were established previously and the MG63 stable cells were established using the same method^15^. U2-OS cells stably knocking down TRIM21 and its control cells were named as sh-TRIM21#1, sh-TRIM21#2, and sh-NC as previously described^16^.

### Co-immunoprecipitation

The co-immunoprecipitation (co-IP) assay was performed as described previously^18^. Briefly, 1 mg of total protein from the lysate was mixed with 2 μg of indicated primary antibodies. The immune complexes were separated by western blotting. These experiments were repeated three times.

### Immunofluorescence assays

Immunofluorescence (IF) assay was performed to determine the subcellular localization of the indicated proteins as described previously^19,20^. To observe the subcellular localization of GFP-ANXA2 in living cells, the cells cultured on coverslip-bottomed small chamber were transfected with GFP-ANXA2 and then mounted onto the stage of LSM700 microscope equipped with a temperature-controlled and CO_2_-controlled small incubator as described previously^21^.

### ELISA kit for alkaline phosphatase

Cultured cells were lysed with lysis buffer A (20 mM Tris pH7.5, 150 mM NaCl, 1% Triton X-100, sodium pyrophosphate, β-glycerophosphate, EDTA, Na_3_VO_4_, leupeptin, and 1% protease inhibitor cocktail (Roche)). Alkaline phosphatase (ALP) activity of the lysates (50 ng/well) was examined by ELISA kit (YEASEN, China) according to the manufacturer’s protocol. Absorbance at 450 nm was measured using a spectrophotometric microplate reader (ELX800, BioTek Instrument, USA). These experiments were repeated three times.

### Gene expression by qRT-PCR

Total RNA was purified and the quantitative real-time PCR (qRT-PCR) assays were performed as described previously^19^. The following primers were used: TRIM21, 5′- TGGACAATTTGGTTGTGGAA-3′ (forward), 5′-ACCATGCCAGCCTCATAGTC-3′ (reverse); ALP, 5′-GACAAGAAGCCCTTCACTGC-3′ (forward), 5′-AGACTGCGCCTGGTAGTTGT-3′ (reverse); RUNX2, 5′-CGGAATGCCTCTGCTGTTAT-3′ (forward), 5′-TGGGGAGGATTTGTGAAGAC-3′ (reverse); ANXA2, 5′-CAGGATATTGCCTTCGCCTACCAG-3′ (forward), 5′-GCGTCATACTGAGCAGGTGTCTTC-3′ (reverse); p62, 5′-GCCTCTGGTTCTGACACTTT-3′ (forward), 5′-GGTGAGGTGGAAGGCATTTA-3′ (reverse); ACTB (the control), 5′-ACGTGGACATCCGCAAAG-3′ (forward), 5′-GACTCGTCATACTCCTGCTTG-3′ (reverse). These experiments were repeated three times.

### Subcellular fractionation assay

For the preparation of nuclear, cytoplasmic, and membrane-enriched proteins, Minute^TM^ Plasma Membrane Protein Isolation and Cell Fractionation Kit (SM-005, Invent Biotechnologies, Eden Prairie, USA) was applied according to the manufacturer’s instructions. Then, the western blotting assay was used to analyze the protein expression in different fractionations. These experiments were performed three times.

### Western blotting

Western blotting was performed as described previously^18,19^. Three independent experiments were performed and the relative quantitative expression was analyzed by Image J software (NIH, USA).

### Tissue microarrays

The expressions of TRIM21 and ANXA2 were analyzed using two consecutively numbered commercial tissue microarrays (TMAs) of OS (OS804C, Alenabio, Xi’an, China), which includes 80 sections of OS tissue samples and every 2 sections coming from one OS case. The company stated that all human tissues are collected under Institutional Review Board and Health Insurance Portability and Accountability Act (HIPAA)-approved protocols. The immunohistochemical staining was performed in Shanghai Outdo Biotech (China) as described previously^18^. Two slides were stained with the primary antibodies against TRIM21 (1 : 200 dilution) and ANXA2 (1 : 500 dilution). Histologic slides were reviewed by two experienced pathologists without knowledge of the relevant information of the patients as reported previously^18^. Briefly, the intensity of protein staining was scored as 0 (negative), 1 (weak), 2 (moderate), 3 (stronger), or 4 (strongest). The percentage of positive cells was scored as 0 (<10%), 1 (11–40%), 2 (41–60%), 3 (61–80%), and 4 (>80%). These two scores were then multiplied. Correlation between the expression of TRIM21 and ANXA2 was estimated using Spearman’s rank correlation analysis.

### Statistical analysis

All values were shown as the means ± SEM of three independent experiments. Statistical analyses were calculated using Student’s *t*-test and the statistical significance was defined as *p* < 0.05.

## Results

### TRIM21 induces autophagy of OS cells

**Fig. 1:**
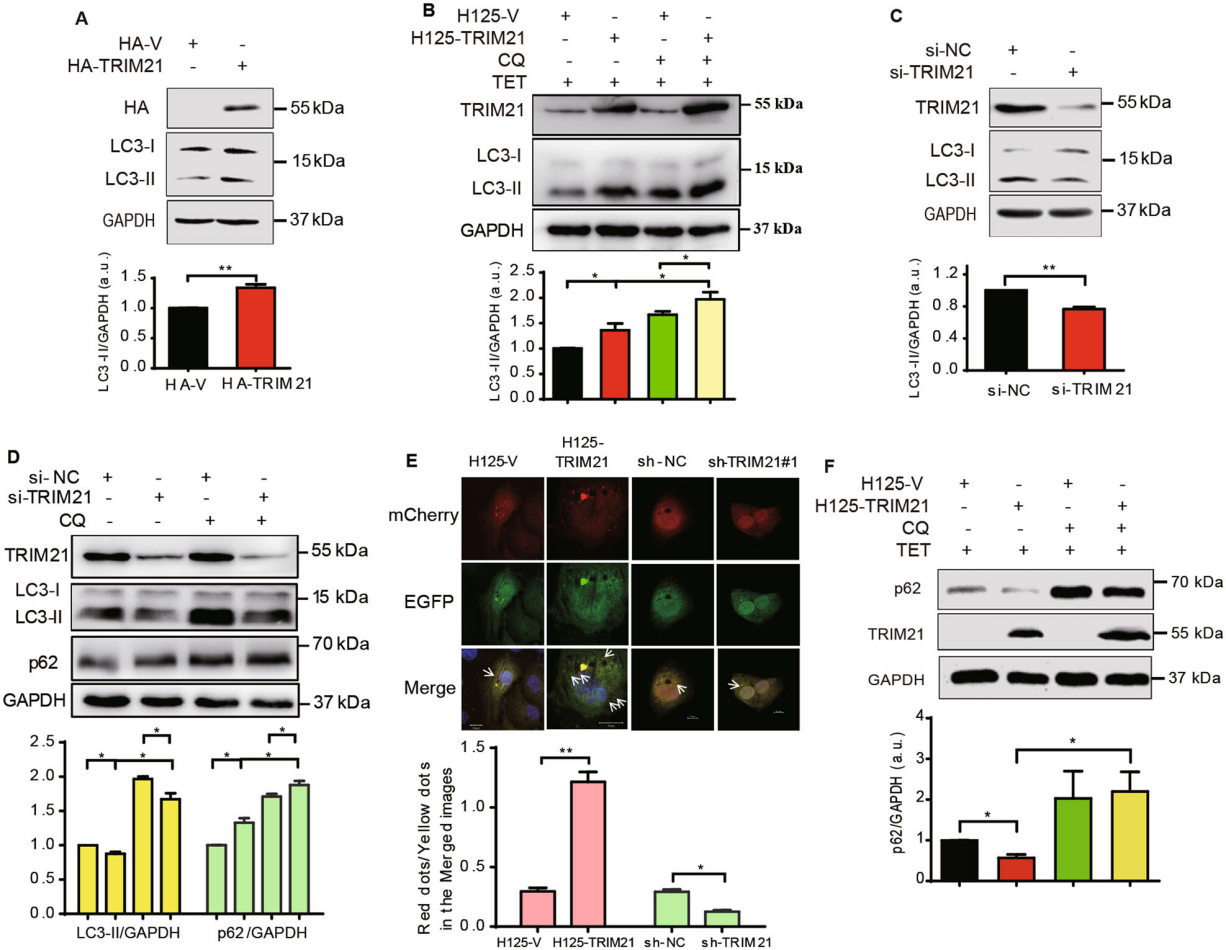
The effects of TRIM21 on OS cell autophagy. **A** OS cells transfected with HA-TRIM21 or HA-vector were collected for western blotting assay. **B** U2-OS cells stably expressing H125-TRIM21 or control H125-V were treated with TET (10 μg/ml, 12 h) or in combinations with CQ (5 μM, 2 h) as indicated. **C** OS cells transfected with si-TRIM21 or si-NC (control) were collected for western blotting assay. **D** U2-OS cells were transfected with si-TRIM21 along with its control and treated with CQ (5 μM, 2 h) as indicated. Then, the cells of **A**–**D** were collected for western blotting assay with the indicated antibodies. The corresponding quantitative analyses of LC3-II/GAPDH or p62/GAPDH were shown in their bottom panels, respectively (*n* = 3, SEM). **E** U2-OS cells stably expressing H125-TRIM21 or control H125-V were transfected with the mCherry-EGFP-LC3 plasmid and treated with TET (10 μg/ml, 12 h) to induce the expression of TRIM21. U2-OS cells stably knocking down TRIM21 (sh-TRIM21#1) or control sh-NC were transfected with the mCherry-EGFP-LC3 plasmid. Then the cells were subjected to IF assay. DAPI staining was used to mark the nucleus. Scale bar: 10 μm. Arrows: the red or yellow dots (upper panel). Lower panel: quantitative analysis of red-only dots/yellow dots of the merged images in 60 cells. **F** U2-OS cells stably expressing H125-TRIM21 or control H125-V were treated with TET (10 μg/ml, 12 h) or in combinations with CQ as indicated. Then the cells were performed western blotting assay. Quantitative analysis of the p62/GAPDH ratio was shown in the bottom panel (*n* = 3, SEM). *p < 0.05, **p < 0.01. All data were representative of three independent experiments.

TRIM21 has been reported to be involved in regulating autophagy of immune cells^7^, yet its role in OS autophagy is largely unknown. We first determined whether TRIM21 regulated the expression of LC3-II, an autophagy marker of autophagosomes^3^. As shown in Fig. 1A, B, overexpression of HA-TRIM21 promoted the expression of LC3-II. Autophagy inhibitor CQ causes LC3-II accumulation, due to its prevention of autophagosome–lysosome fusion as reported previously^22^. TRIM21 overexpression caused a further increase of CQ-mediated (5 μM) LC3-II accumulation. In contrast, knockdown of TRIM21 reduced the accumulation of LC3-II in both the control and CQ-stimulated conditions (Fig. 1C, D). Next, IF assay using a pH-sensitive mCherry-EGFP-LC3^23^ also showed that overexpression of TRIM21 remarkably increased the ratio of red-only to yellow dots, whereas knockdown of TRIM21 attenuated this ratio (Fig. 1E), suggesting that TRIM21 is essential for promoting the formation of autophagosomes and its delivery to lysosomes.

Then, we tested whether TRIM21 participated in the degradation of p62, an autophagy receptor, which can be degraded together with cargos^3,24^. Overexpression of TRIM21 significantly diminished the p62 expression, whereas, its expression level was restored by CQ (Fig. 1F). In contrast, TRIM21 knockdown inhibited the degradation of p62, similar to the effect of CQ (Fig. 1D). These results collectively suggest that TRIM21 plays a crucial role in the induction of OS cell autophagy.

### ANXA2 is identified as a novel interacting protein of TRIM21, and it counteracts on TRIM21-mediated autophagy

**Fig. 2:**
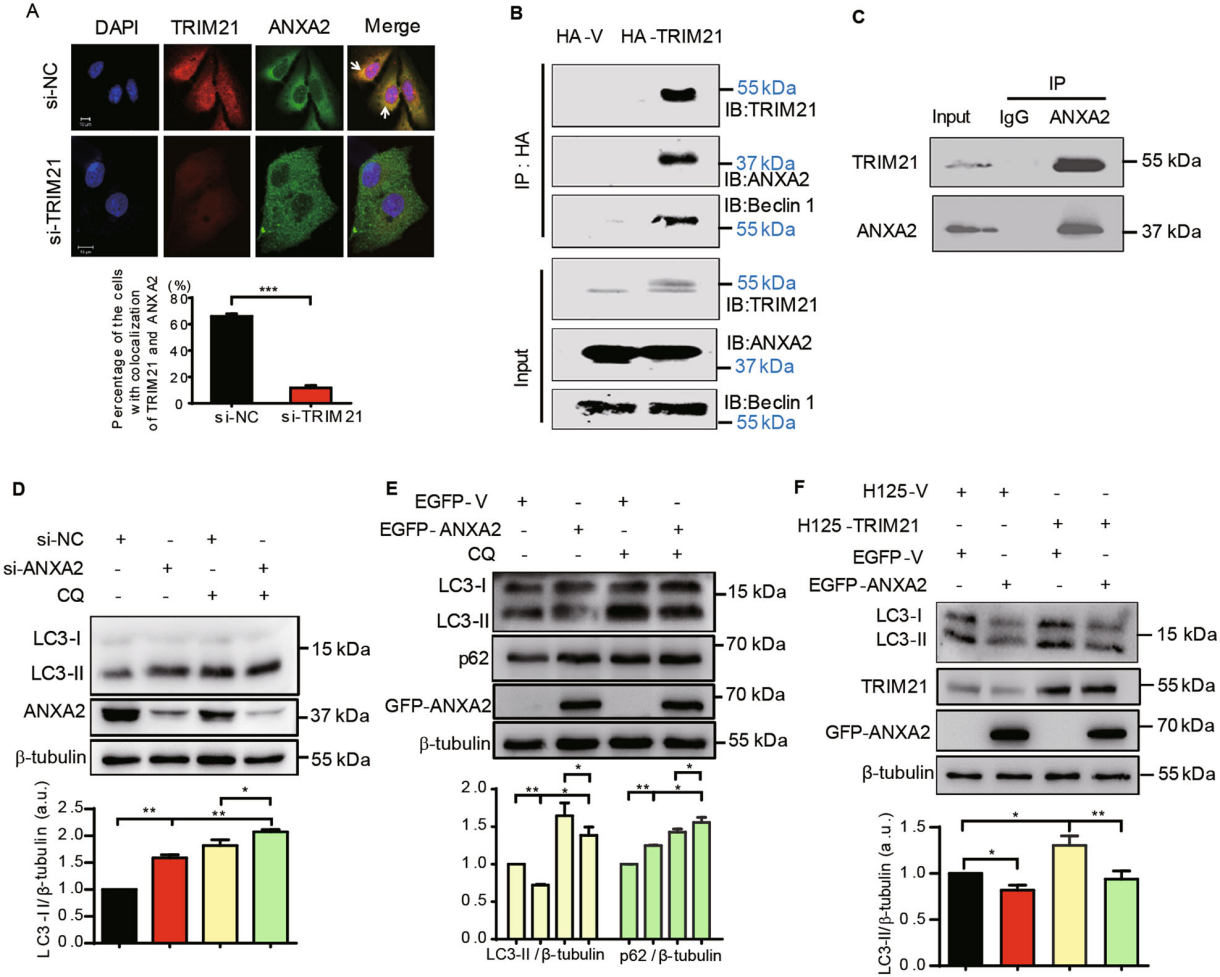
ANXA2 interacts with TRIM21 and counteracts on TRIM21-induced autophagy. **A** U2-OS cells transfected with si-NC or si-TRIM21 were performed the IF assay, then the colocalization of TRIM21 and ANXA2 was observed using a confocal microscope (upper panel). The lower is the percentage of cells exhibiting colocalization of TRIM21 with ANXA2 of 80 cells. Scale bar: 10 μm. **B** U2-OS cells transfected with HA-vector or HA-TRIM21 were used to perform co-IP assay with HA antibody. The immune complexes of the HA antibody were analyzed by immunoblotting with the indicated antibodies. **C** U2-OS cells were used to perform co-IP assay with ANXA2 antibody. U2-OS cells transfected with si-ANXA2, si-NC (**D**), or EGFP-ANXA2, EGFP-V (**E**) in combination with CQ (5 μM, 2 h) treatment as indicated were performed western blotting assay. The corresponding quantitative analyses of LC3-II/β-tubulin or p62/β-tubulin ratios were shown in their lower panels, respectively (*n* = 3, SEM). **F** U2-OS cells stably expressing H125-TRIM21 or control H125-V were transfected with EGFP-ANXA2 or EGFP-vector and treated with TET (10 μg/ml, 12 h) to perform western blotting assay. LC3-II/β-tubulin was shown in its lower panel (*n* = 3, SEM). *p < 0.05, **p < 0.01, ***p < 0.001. ns: not significant. All data were representative of three independent experiments.

Next, we sought to explore the underlying mechanism of TRIM21-mediated OS cell autophagy. Previously, we had identified a series of interacting proteins of TRIM21 in the U2-OS cells^15^. Among them, ANXA2 exhibited the highest unique peptide number and sequence coverage rate (Supplementary Table S1). ANXA2 is a calcium- and phospholipid-dependent membrane-binding protein that has been reported to regulate osteogenic differentiation, autophagy, and OS tumorigenesis^25–28^; we thus hypothesized that ANXA2 play a role in TRIM21-regulated autophagy via protein–protein interaction. Notably, a cytoplasmic colocalization of TRIM21 and ANXA2 was observed in U2-OS cells, with a marked decrease when TRIM21 was knocked down (Fig. 2A). Co-IP assay further validated that ANXA2 was precipitated by the HA antibody in the HA-TRIM21 group but not in control cells (Fig. 2B). Similarly, endogenous TRIM21 and ANXA2 were coprecipitated by the ANXA2 antibody (Fig. 2C), suggesting that TRIM21 and ANXA2 interacted with each other. Interestingly, another autophagy-related protein, Beclin 1, was also identified in the same immune complex of HA-TRIM21 (Fig. 2B), indicating that TRIM21 might also participate in the early stage of autophagy through interacting with Beclin 1. Next, we explored the role of ANXA2 in TRIM21-mediated OS cells autophagy. ANXA2 knockdown significantly increased the LC3-II expression either in the presence or absence of CQ (Fig. 2D). Conversely, overexpression of EGFP-ANXA2 reduced the expression of LC3-II, while increased the accumulation of p62, which was also observed when combined with CQ treatment (Fig. 2E). Furthermore, we did find that overexpression of ANXA2 compromised the induction of LC3-II by enforced TRIM21 expression (Fig. 2F). These results together suggest that ANXA2 negatively regulates OS cell autophagy induced by TRIM21.

### TRIM21 promotes the translocation of ANXA2 toward cell membrane

**Fig. 3:**
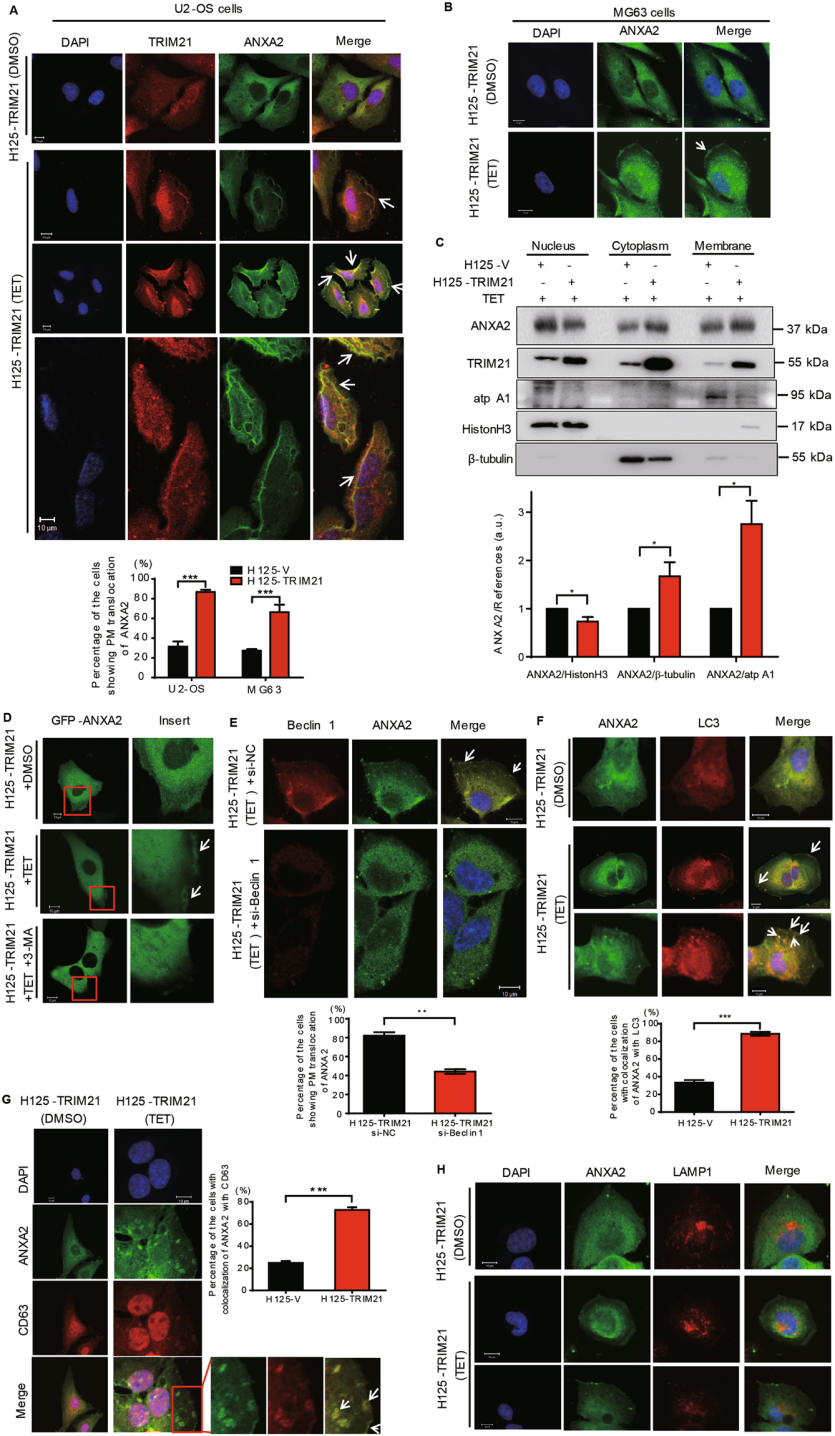
TRIM21 promotes the translocation of ANXA2 toward PM. **A** U2-OS cells stably expressing H125-TRIM21 were treated with DMSO or TET (10 μg/ml) to induce the overexpression of TRIM21 and then carried out IF assay (upper panel). Percentage of the cells showing PM translocation of ANXA2 of 90 cells in **A** and **B** (lower panel). **B** MG63 cells stably expressing H125-TRIM21 were treated with DMSO or TET (10 μg/ml) and then carried out IF assay. **C** U2-OS cells stably expressing H125-TRIM21 or H125-V were treated with TET (10 μg/ml) and then performed subcellular fractionation assay to isolate the nuclear, cytoplasmic and membrane fractions. The western blotting assay was carried out with the indicated antibodies. The quantitative analyses of ANXA2/references (including ANXA2/HistonH3 in the nucleus, ANXA2/β-tubulin in the cytoplasm and ANXA2/atp A1 at the membrane) were shown in the lower panel (*n* = 3, SEM). **D** U2-OS cells stably expressing H125-TRIM21 were treated with DMSO or TET and added with 3-MA (10 nM) to inhibit autophagy as indicated. The living cells were used to observe the location of GFP-ANXA2. **E** U2-OS cells stably expressing H125-TRIM21 were transfected with si-NC or si-Beclin 1 and then treated with TET for IF assay using the ANXA2 and Beclin 1 antibodies (upper panel). Lower panel: the percentage of cells showing PM translocation of ANXA2 of 60 cells. Upper panel: U2-OS cells stably expressing H125-TRIM21 were treated with DMSO or TET to perform IF assay using ANXA2 and LC3 antibodies (**F**), ANXA2 and CD63 antibodies (**G**), and ANXA2 and LAMP1 antibodies (**H**). Percentages of the cells with colocalization of ANXA2 with LC3 or ANXA2 with CD63 of 60 cells were shown in their lower or right panels, respectively. Scale bar: 10 μm. *p < 0.05, **p < 0.01, ***p < 0.001. All data were representative of three independent experiments.

Next, we investigate whether TRIM21, as an E3 ubiquitin ligase^29^, may regulate the expression of ANXA2. Our results showed that neither overexpression nor knockdown of TRIM21 had visible effects on the protein and mRNA expression of ANXA2 (Supplementary Fig. S1). As the subcellular trafficking of ANXA2 is another major form of ANXA2 regulation, we then tested whether TRIM21 plays a role in regulating its localization. As shown in Fig. 3A, B, stable overexpression of TRIM21 remarkably increased the translocation of ANXA2 toward PM in both the U2-OS and MG63 cells. Consistently, the subcellular fractionation assay also demonstrated that overexpression of TIRM21 upregulated the expression of ANXA2 at the cytoplasm and cell membrane, while decreased its level within the nucleus (Fig. 3C).

According to a recent study, ANXA2 was incorporated into autophagosomes after interferon-γ (IFN-γ) stimulation, followed by the fusion with multivesicular bodies (MVBs) for PM, but not with the lysosome^30^. We then investigated whether the trafficking of ANXA2 was related to the autophagy induced by TRIM21. As shown in Fig. 3D, TRIM21 overexpression resulted in obvious trafficking of GFP-ANXA2 to the PM in living cells (arrows). However, the addition of 3-MA, an upstream inhibitor of autophagy, significantly inhibited ANXA2 localization at the PM. Furthermore, knockdown of Beclin 1 significantly decreased the trafficking of ANXA2 to the PM which mediated by the TRIM21 overexpression (Fig. 3E). IF assay further confirmed the colocalization of ANXA2 with LC3 upon TRIM21 overexpression (Fig. 3F), indicating that ANXA2 was incorporated into the autophagosomes. Next, we observed the colocalization of ANXA2 with CD63 (a marker of MVBs) in the TRIM21-overexpressed cells; however, no colocalization of ANXA2 and LAMP1 (a lysosomal marker) was observed, indicating that ANXA2, after incorporating into the autophagosomes, was subsequently inserted into the MVBs for PM localization, but not for lysosomes (Fig. 3G, H). These results suggest that the translocation of ANXA2 toward PM is dependent on TRIM21-induced autophagy and this translocation might favor TRIM21-induced autophagy.

### Co-expression of TRIM21 and ANXA2 at the PM in OS tissues

**Fig. 4:**
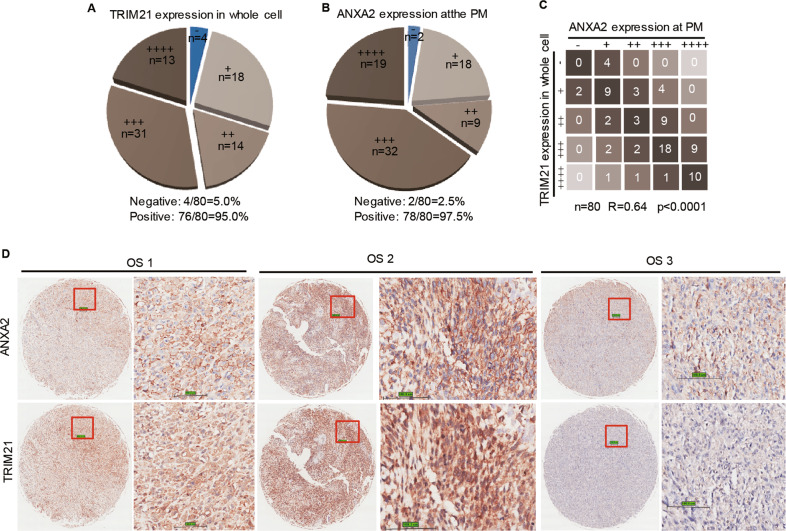
The expression of TRIM21 is correlated with ANXA2 expression at the PM in human OS tissues. Pie chart shows the grades of the expression levels of TRIM21 (**A**) and ANXA2 at the PM (**B**) in TMAs of human OS tissues. **C** Statistical analysis of the correlation expressions of TRIM21 and ANXA2 at the PM. *P*-value was calculated with Spearman’s rank correlation analysis. “−”: negative expression; “+”: low expression; “++”: medium expression; “+++”: higher expression, “++++”: highest expression. **D** Three representative cases of the correlation analysis of TRIM21 and ANXA2 expressions in human OS TMAs. Scale bar: 100 μm.

Having established a positive correlation of TRIM21 with the PM localized ANXA2, we next sought to extend this finding into clinical context by using the TMAs of OS tissues. As shown in Fig. 4A, B, 95% of OS tissues positively expressed TRIM21 and 97.5 % positively expressed ANXA2 at the PM. Moreover, a positive correlation between TRIM21 expression in the whole-cell and ANXA2 expression at the PM was established, with a statistical significance (*R* = 0.64, *P* < 0.0001, Fig. 4C). Three representative cases were shown in Fig. 4D.

### TRIM21 disrupts the ANXA2-TFEB complex and promotes the nuclear translocation of TFEB

**Fig. 5:**
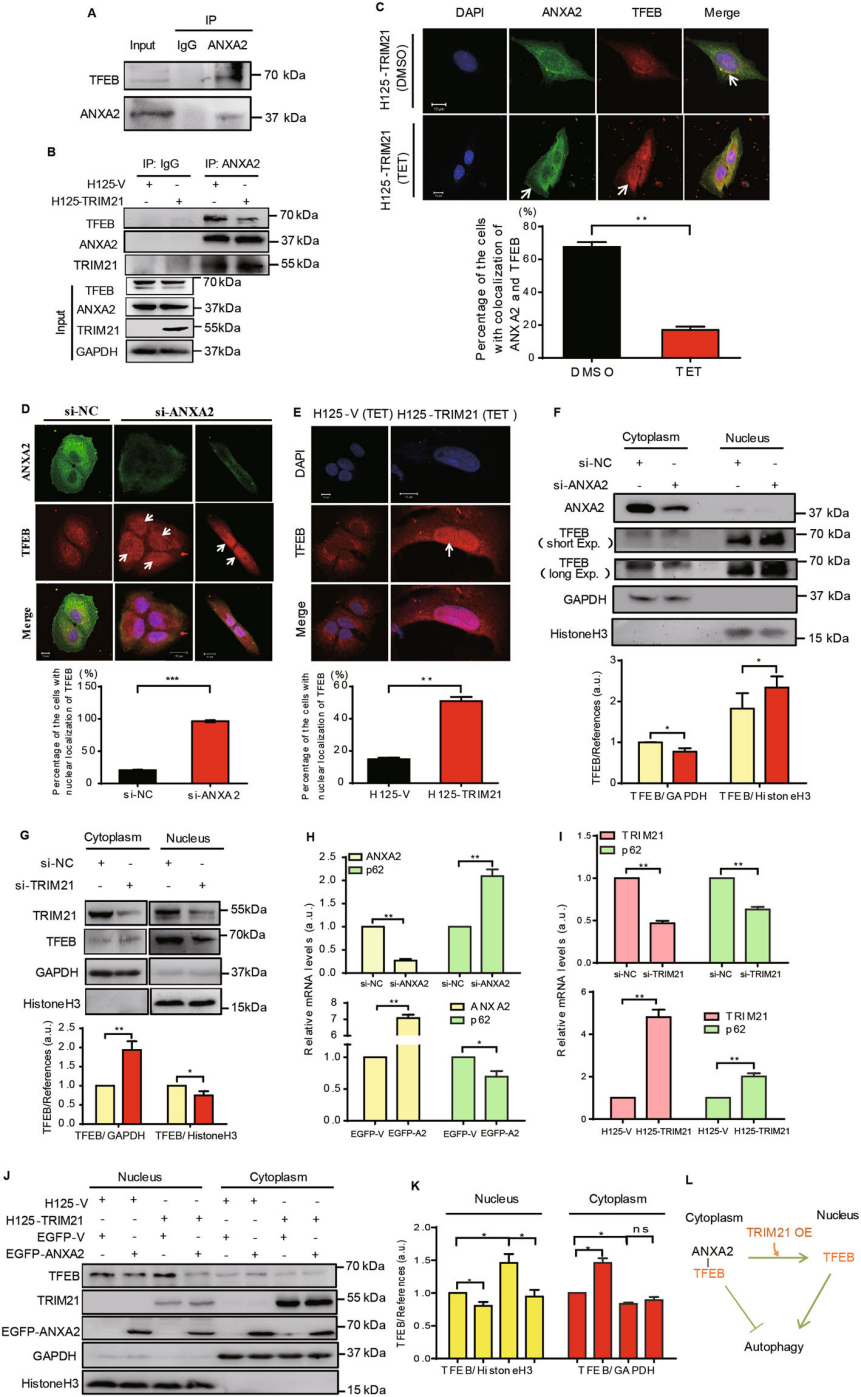
TRIM21 separates the ANXA2-TFEB complex and thus facilitates the autophagy in OS cells. **A** U2-OS cells were used to perform co-IP assay with ANXA2 antibody. **B** U2-OS cells stably expressing H125-V or H125-TRIM21 were used to perform co-IP assay with IgG and ANXA2 antibodies after TET (10 μg/ml) treatment. **C** U2-OS cells stably expressing H125-TRIM21 were treated with DMSO or TET and then performed IF using ANXA2 and TFEB antibodies (Upper panel). (Lower panel) Percentage of the cells with colocalization of ANXA2 and TFEB in 50 cells. **D** U2-OS cells were transfected with si-NC or si-ANXA2 and then performed IF using ANXA2 and TFEB antibodies (upper panel). Lower panel: percentage of the cells with nuclear localization of TFEB in 80 cells. Arrows: nucleus. **E** U2-OS cells stably expressing H125-TRIM21 or H125-V were treated with TET and then performed IF using the TFEB antibody (Upper panel). Lower panel: Percentage of the cells with nuclear localization of TFEB in 80 cells. Arrow: nucleus. U2-OS cells were transfected with si-ANXA2 (**F**) or si-TRIM21 (**G**) and then performed subcellular fractionation assay to isolate the nuclear and cytoplasmic fractions (Upper panel). The corresponding quantitative analyses of TFEB/References were shown in their lower panels, respectively (*n* = 3, SEM). **H**, **I** U2-OS cells transfected with si-ANXA2, EGFP-ANXA2 or si-TRIM21, or the stable U2-OS cells expressing H125-TRIM21 or H125-V with TET treatment as indicated to perform qRT-PCR assays to analyze the mRNA expression of p62, ANXA2, or TRIM21 (*n* = 3, SEM). **J** U2-OS cells stably expressing H125-TRIM21 or H125-V were transfected with EGFP-ANXA2 as indicated and treated with TET to perform the subcellular fractionation assay using the indicated antibodies. **K** The quantitative analyses of TFEB/References in the nucleus and cytoplasm in **J** (*n* = 3, SEM). **L** A supposed association of TRIM21, ANXA2, and TFEB. Scale bar: 10 μm. *p < 0.05, **p < 0.01, ***p < 0.001. All data were representative of three independent experiments.

In lung epithelial cells, ANXA2 has been reported to interact with TFEB, a master transcription factor for autophagy, thus impeding autophagy through cytoplasmic retention and inactivation of TFEB^27,31–33^. Consistent with this, our co-IP assay did confirm that TFEB interacted with ANXA2 in the U2-OS cells (Fig. 5A). Of significance, overexpression of TRIM21 diminished the interaction between ANXA2 and TFEB, while favoring ANXA2 and TRIM21 interaction (Fig. 5B). A similar result was observed by IF assay, in which overexpression of TRIM21 reduced the colocalization of ANXA2 and TFEB in the cytoplasm (Fig. 5C). Instead, an increase of PM localization of ANXA2 and nuclear localization of TFEB was found (Fig. 5C). Thus, these results collectively suggest that TRIM21 impedes the cytoplasmic association between ANXA2 and TFEB, and that the translocation of ANXA2 towards PM might contribute to the release of TFEB from the complex of ANAX2-TFEB.

We then evaluated the impacts of ANXA2 and TRIM21 on the localization of TFEB. As shown in Fig. 5D, E, either knockdown of ANXA2 or overexpression of TRIM21 promoted the nuclear translocation of TFEB. Similarly, the increased nuclear translocation of TFEB was confirmed using the subcellular fractionation assay upon ANXA2 knockdown and the diminished nuclear translocation of TFEB was found upon TRIM21 knockdown (Fig. 5F, G). These results indicate that TRIM21 promotes the nuclear translocation of TFEB, whereas ANXA2 has the opposite effect. As TFEB can upregulate autophagy-related genes such as *p62/SQSTM1*^31^, we explored the role of ANXA2 and TRIM21 in regulating *p62* mRNA expression. As shown in Fig. 5H, ANXA2 knockdown caused a significant increase in *p62* mRNA expression, while EGFP-ANXA2 overexpression decreased it. Interestingly, we demonstrated that TRIM21 played an opposite role in regulating p62 (Fig. 5I). These results indicate that ANXA2 and TRIM21 affect the mRNA expression of p62 through regulating the TFEB translocation. Furthermore, overexpression of EGFP-ANXA2 lessened the nuclear expression of TFEB, whereas overexpression of TRIM21 augmented the nuclear expression of TFEB. This augments of nuclear TFEB was compromised by the overexpression of EGFP-ANXA2 (Fig. 5J, K). These results suggest that ANXA2 suppresses the nuclear translocation of TFEB induced by TRIM21 and thus triggering OS cell autophagy (Fig. 5L).

### TRIM21 inhibits osteogenic differentiation of OS cells by inducing autophagy

Autophagy has been shown to positively regulate osteogenic differentiation of osteoblast^34–37^, we then hypothesized that TRIM21 might regulate OS cell differentiation through the induction of autophagy. Serum starvation caused an increase of LC3-II, accompanied by a downregulation of RUNX2 (Supplementary Fig. S2a, b), a master osteogenic marker for osteoblast and OS^38^. Conversely, inhibition of autophagy with CQ elevated the expression of RUNX2, which was re-decreased by the protein synthesis inhibitor CHX (Suppleentary Fig. S2c–f). These results suggested that autophagy might inhibit the osteogenic differentiation of OS cells through a transcriptional regulation rather than autophagy degradation of RUNX2 (Supplementary Fig. S2g). Next, we determined the expression of TRIM21 in a series of OS cells with different differentiation status. It has been reported that the degrees of differentiation in OS cells MG63, U2-OS, Saos-2 were gradually increased^39–42^. In line with this, our results confirmed that the levels of the differentiation markers RUNX2 and ALP were gradually increased in the MG63, U2-OS, and Saos-2 cells (Supplementary Fig. S2h, i). Interestingly, the degrees of autophagy and expression of TRIM21 was gradually decreased in these cells. These results suggest that the expression of TRIM21 is negatively correlated with OS differentiation.

**Fig. 6:**
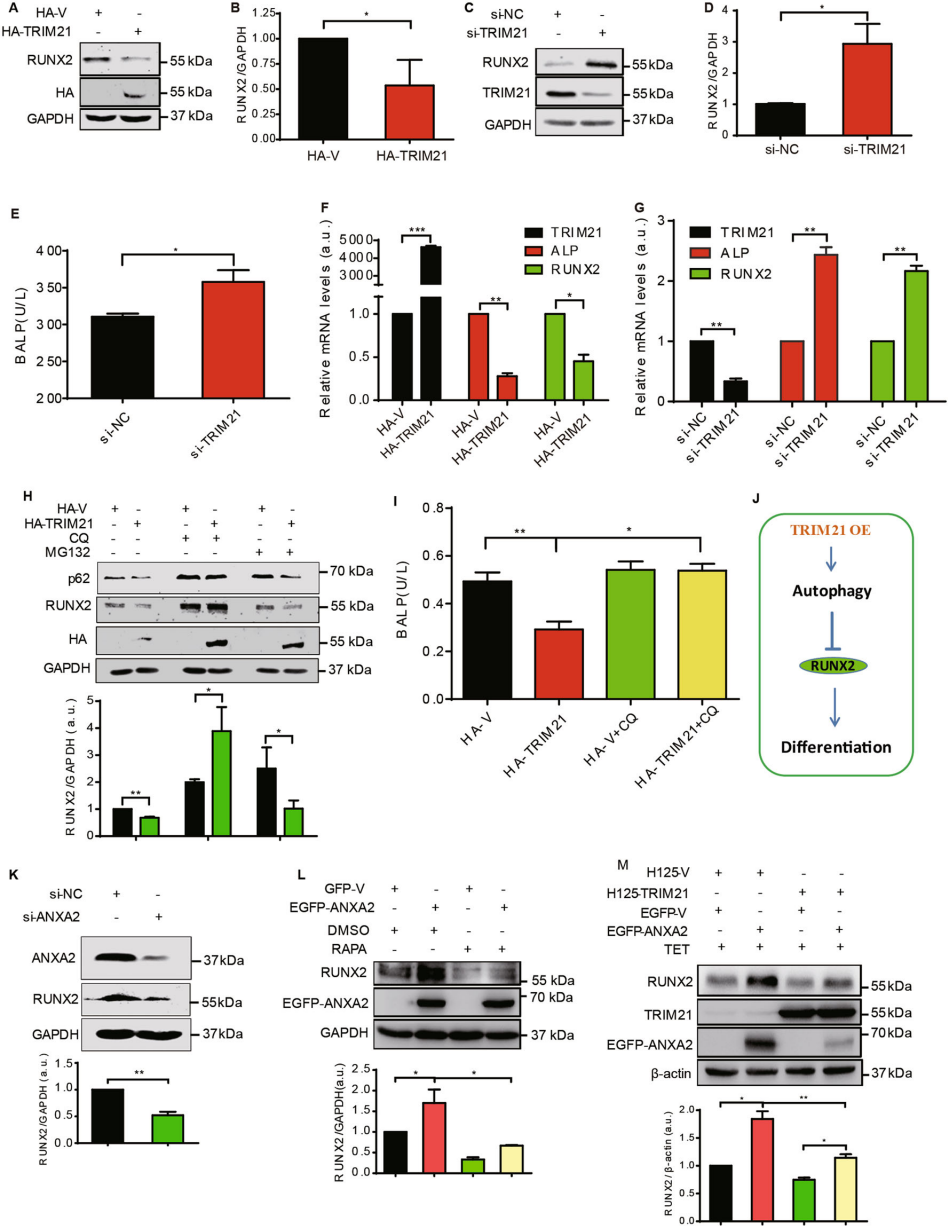
TRIM21 inhibits OS cell differentiation in an autophagy-dependent manner. **A**–**D** OS cells were transfected with HA-TRIM21 (**A**) or siRNA of TRIM21 (**C**) and performed western blotting assay. The corresponding quantitative analyses of the RUNX2/GAPDH ratio were shown in **B** and **D** (*n* = 3, SEM). **E** U2-OS cells were transfected with siRNA of TRIM21 and harvested for detection of ALP activity using ELISA assay (*n* = 3, SEM). U2-OS cells were transfected with HA-TRIM21 (**F**) or TRIM21 siRNA (**G**), respectively, and collected for detection of the expression of TRIM21, ALP, and RUNX2 by qRT-PCR assay (*n* = 3, SEM). **H** U2-OS cells were transfected with HA-TRIM21 and treated with CQ and MG132 (100 nM) for 24 h. The cells were harvested and performed western blotting assay with the indicated antibodies. The ratio of RUNX2/GAPDH was shown in its lower panel (*n* = 3, SEM). **I** U2-OS cells were transfected with HA-TRIM21 or HA-vector and harvested for detection of ALP activity using ELISA assay (*n* = 3, SEM). **J** A model of the relationship between TRIM21 and differentiation. TRIM21 OE: TRIM21 overexpression. **K** OS cells transfected with si-NC or si-ANXA2 were performed western blotting assay (Upper panel). RUNX2/GAPDH was shown in its lower panel (*n* = 3, SEM). **L** U2-OS cells were transfected with EGFP-V or EGFP-ANXA2 and treated with RAPA (1.5 μM, 24 h) as indicated to perform western blotting assay (upper panel). Lower panel: the ratio of RUNX2/GAPDH (*n* = 3, SEM). **M** U2-OS cells stably expressing H125-TRIM21 or H125-V were transfected with EGFP-ANXA2 or EGFP-vector and treated with TET as indicated to perform western blotting assay. RUNX2/β-actin was shown in its lower panel (*n* = 3, SEM). *p < 0.05; **p < 0.01. All data were representative of three independent experiments.

We then explored the implication of TRIM21 in OS differentiation. As shown in Fig. 6A, B, overexpression of HA-TRIMA21 significantly reduced the level of RUNX2, while TRIM21 knockdown increased RUNX2 expression and ALP activity (Fig. 6C–E). Moreover, overexpression of HA-TRIM21 significantly reduced the expression of RUNX2 and ALP at the transcriptional levels (Fig. 6F). Inversely, TRIM21 knockdown significantly enhanced the transcriptional levels of RUNX2 and ALP (Fig. 6G). Thus, TRIM21 inhibits OS cell differentiation marked with the negative regulation of the transcriptional expressions of RUNX2 and ALP.

To examine whether the effect of TRIM21 on OS cell differentiation was dependent on the TRIM21-induced autophagy, the autophagy inhibitor CQ was applied. As shown in Fig. 6H, overexpression of HA-TRM21 diminished the level of RUNX2; however, it was restored upon the treatment of CQ, but not the proteasome inhibitor, MG132 (Fig. 6H). Similarly, the decline of ALP activity induced by TRIM21 overexpression was also restored by the CQ (Fig. 6I). Collectively, these results indicate that autophagy is a prerequisite for the inhibitory role of TRIM21 in OS cell differentiation (Fig. 6J). Next, we tested whether ANXA2 was involved in the regulation of OS cell differentiation mediated by TRIM21. In agreement with the previous report^25^, knockdown of ANXA2 decreased RUNX2 expression (Fig. 6K), while overexpression of ANXA2 increased its expression level, which was suppressed by the autophagy activator RAPA (Fig. 6L). Furthermore, ANXA2 overexpression reversed the downregulated trend of RUNX2 induced by TRIM21 overexpression (Fig. 6M), suggesting that ANXA2 counteracted TRIM21-mediated differentiation inhibition. In addition, TFEB knockdown compromised the decreasing trend of RUNX2 induced by TRIM21 overexpression (Supplementary Fig. S3), indicating that TFEB plays a role in TRIM21-regulated RUNX2 expression.

## Discussion

**Fig. 7:**
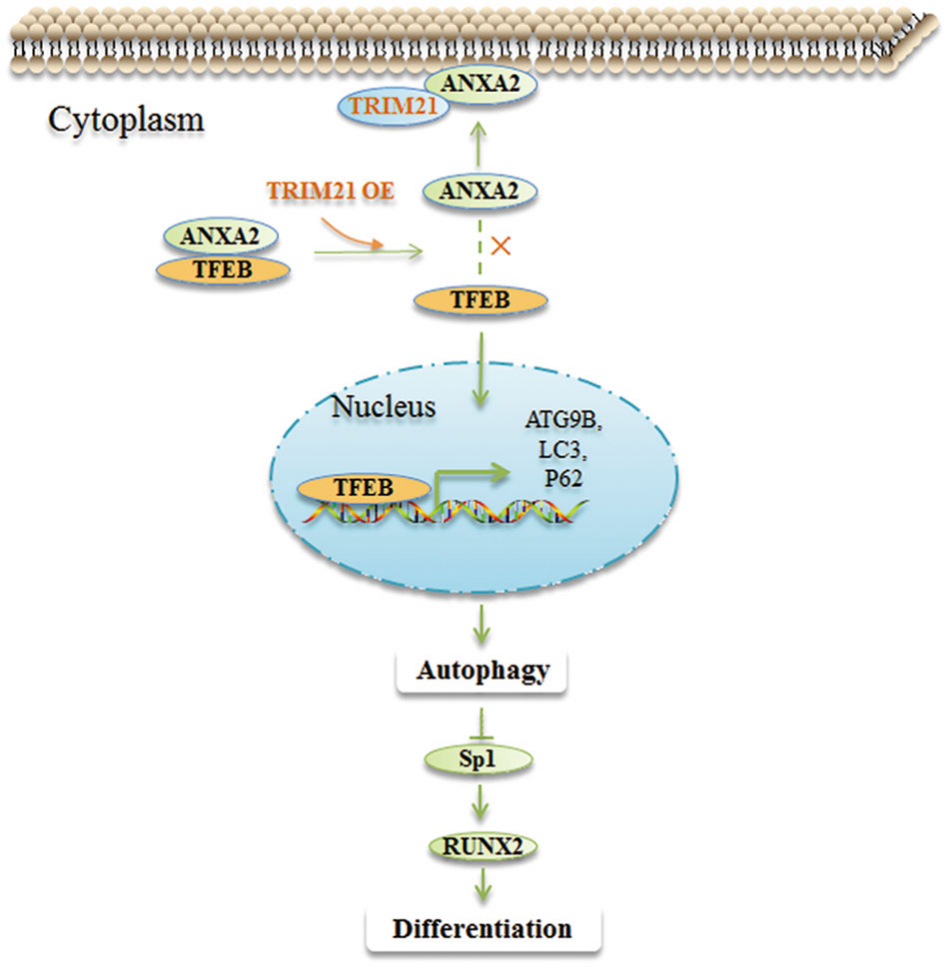
A schematic model of TRIM21 regulating OS autophagy and differentiation by interacting with ANXA2. When TRIM21 is overexpressed in OS cells, it competitively binds to ANXA2 and thus facilitating the translocation of ANXA2 toward PM; this enables the disassociation of TFEB from the ANXA2-TFEB complex, allowing TFEB entering into the nucleus. Nuclear TFEB promotes the transcription of autophagy-related genes, including *ATG9B*, *LC3*, and *P62*, and inhibits OS differentiation, presumably, through regulating the Sp1/RUNX2 signaling in OS cells. TRIM21 OE: TRIM21 overexpression. ×: disassociation of ANXA2-TFEB complex.

Recently, TRIM21, despite its role as E3 ubiquitin ligases, has been extensively demonstrated to be involved in the regulation of autophagy^7,29^, yet its role in OS cell autophagy remains unclear. In this study, we showed that TRIM21 promoted OS cell autophagy by enhancing the autophagic flux and the degradation of p62 (Fig. 1). A previous study has reported that TRIM21 ubiquitylates p62 and abrogates p62 oligomerization and sequestration of client proteins for autophagic degradation under proteotoxic stress and starvation^43^. In other cases, TRIM21 has been shown to facilitate autophagy by assembling core components of autophagic machinery including ULK1, BECN1, and p62 in the cells responding to IFN-γ stimulation^6,44^. Thus, both the influence of TRIM21 on autophagy and the interplay between TRIM21 and p62 are highly cell-type dependent. ANXA2 was screened and identified as an interesting interaction partner of TRIM21 (Fig. 2) due to its backgrounds in both autophagy and osteoblast differentiation^25–28^. ANXA2 has been demonstrated to not only positively regulate autophagy^45–47^ but also participate in the negative regulation of autophagy by impeding autophagic flux and inducing cytoplasmic retention of TFEB^27^. TFEB upregulates the number of autophagosomes and increases gene expressions of *ATG9B*, *p62/SQSTM1*, *LC3*, *UVRAG*, *WIPI*, *VPS11*, and *VPS18*, the direct targets of TFEB^31^. Here we showed that ANXA2 not only inhibited OS cell autophagy but also antagonized TRIM21-induced autophagy (Fig. 2). Overexpression of TRIM21 partly compromised the inhibitory role of ANXA2 in autophagy through disrupting the ANXA2-TFEB complex, abolishing the cytoplasmic retention of TFEB, and facilitating the nuclear translocation of TFEB (Figs. 2 and 5). Collectively, these results suggest that the TRIM21/ANXA2/TFEB axis is involved in OS cell autophagy (Fig. 7).

The relationships between autophagy and differentiation are still controversial. During osteogenesis and osteoclastogenesis, autophagy is actively induced and required for differentiation^34,35,37,48–51^. Other research showed that pneumolysin (PLY), a major virulence factor of *Streptococcus pneumonia*, inhibits osteoblast differentiation by inducing autophagy^39^. Mechanistically, PLY-induced autophagy downregulates the expression of transcription factor Sp1, essential for differentiation, and subsequently inhibits osteoblast differentiation^39^. Sp1 upregulates RUNX2 and both of them are key transcription factors of osteoblast differentiation^52^. Here, TRIM21-induced autophagy inhibited the expression of RUNX2 (Fig. 6). It is likely that the inhibitory effect of autophagy induced by TRIM21 on the differentiation is through the Sp1/RUNX2 signaling in OS cells (Fig. 7).

ANXA2 has been reported to be implicated in the positive regulation of osteoblastic differentiation^25,26,28,53^. Consistently, our result showed that ANXA2 positively regulated OS differentiation (Fig. 6). ANXA2 is reported to express in the cytoplasm, nucleus, and PM^54^, and able to translocate toward PM under several conditions^55–57^. Herein, we demonstrated that TRIM21 overexpression facilitated the translocation of ANXA2 toward PM (Fig. 3), which might be beneficial for the release of TFEB from its inhibitory complex and thus activating cell autophagy (Fig. 7).

In conclusion, our study provides deep insights into the biological function and molecular mechanism of TRIM21 in OS autophagy and differentiation, which highlights the potential of TRIM21 silencing as a promising therapeutic strategy for OS. In addition, our findings that the TRIM21/ANXA2/TFEB axis is involved in OS cell autophagy and subsequent differentiation, also might lead to a new clue for OS treatment.

## Supplementary information

Table S1

Figure S1

Figure S2

Figure S3

Supplementary Material
