# Supplementary material for: TRIM21-regulated Annexin A2 plasma membrane trafficking facilitates osteosarcoma cell differentiation through the TFEB-mediated autophagy
Source: Cell Death Dis. 2021 Jan 6;12(1):21. doi: 10.1038/s41419-020-03364-2 (PMC7790825; doi:10.1038/s41419-020-03364-2)
Supplement: Supplementary file 1 — Table S1 [file 41419_2020_3364_MOESM1_ESM.docx]

Table S1 The first six proteins interacting with TRIM21 identified by LC-MS/MS.

| Protein IDs | Protein  Descriptions | Gene  names | Unique  Peptides | Sequence coverage [%] | Mol. Weight [kDa] | Protein FDR confidence |
| --- | --- | --- | --- | --- | --- | --- |
| P63104 | 14-3-3 protein zeta/delta | YWHAZ | 3 | 12.6531 | 27.7 | High |
| P26641 | Elongation factor 1-gamma | EEF1G | 2 | 6.40732 | 50.1 | High |
| P17066 | Heat-shock 70 kDa protein 6 | HSPA6 | 2 | 6.06532 | 71 | High |
| P07355 | Annexin A2 | ANXA2 | 6 | 20.9440 | 38.6 | High |
| P60709 | Actin | ACTB | 4 | 16.5333 | 41.7 | High |
| P07900 | Heat shock protein HSP 90-alpha | HSP90AA1 | 2 | 3.2787 | 84.6 | High |

FDR: False Discovery Rate.
