# Supplementary figures and images for: TRIM21-regulated Annexin A2 plasma membrane trafficking facilitates osteosarcoma cell differentiation through the TFEB-mediated autophagy
Source: Cell Death Dis. 2021 Jan 6;12(1):21. doi: 10.1038/s41419-020-03364-2 (PMC7790825; doi:10.1038/s41419-020-03364-2)

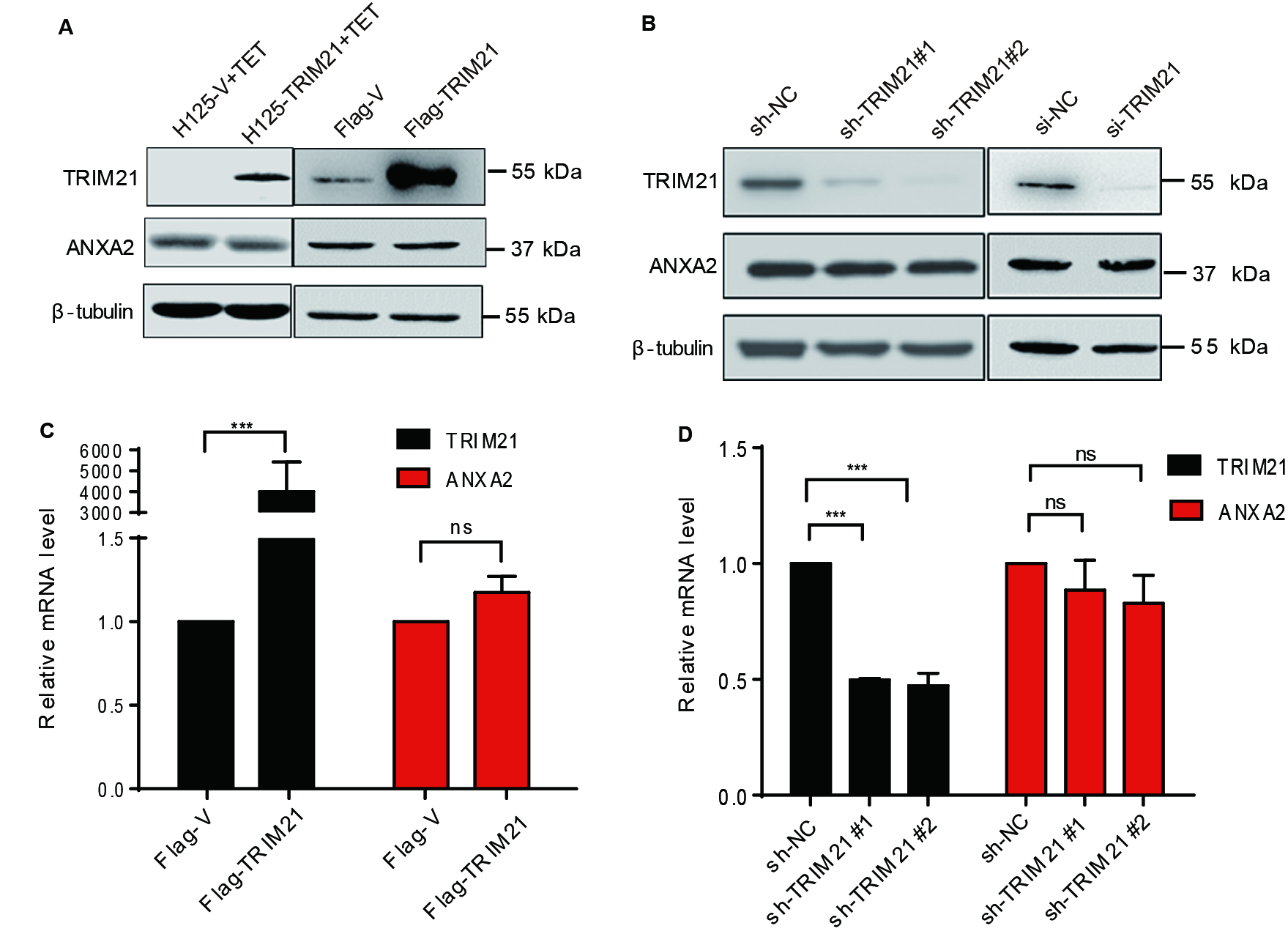

Supplement: Supplementary file 2 — Figure S1 [file 41419_2020_3364_MOESM2_ESM.tif]

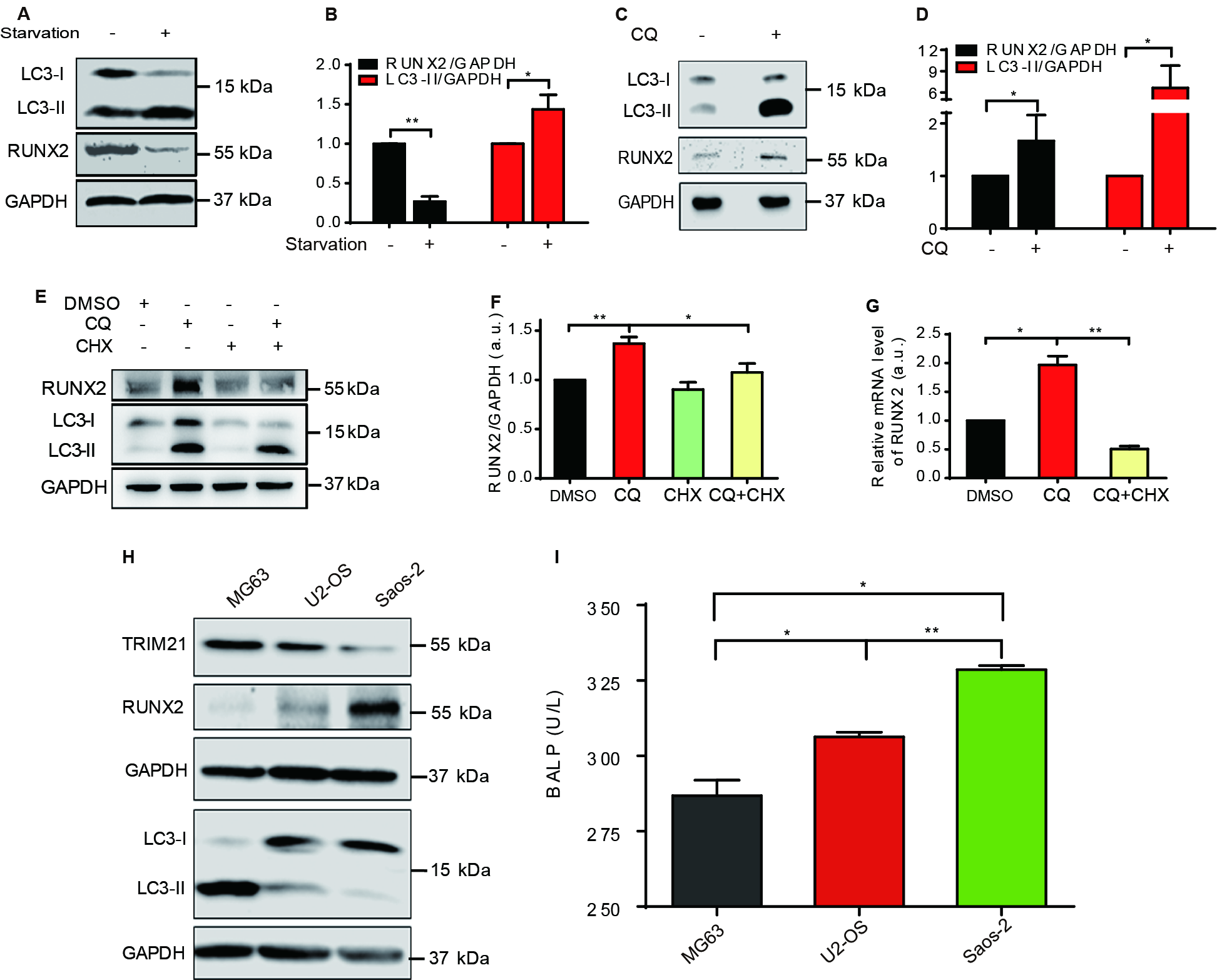

Supplement: Supplementary file 3 — Figure S2 [file 41419_2020_3364_MOESM3_ESM.tif]

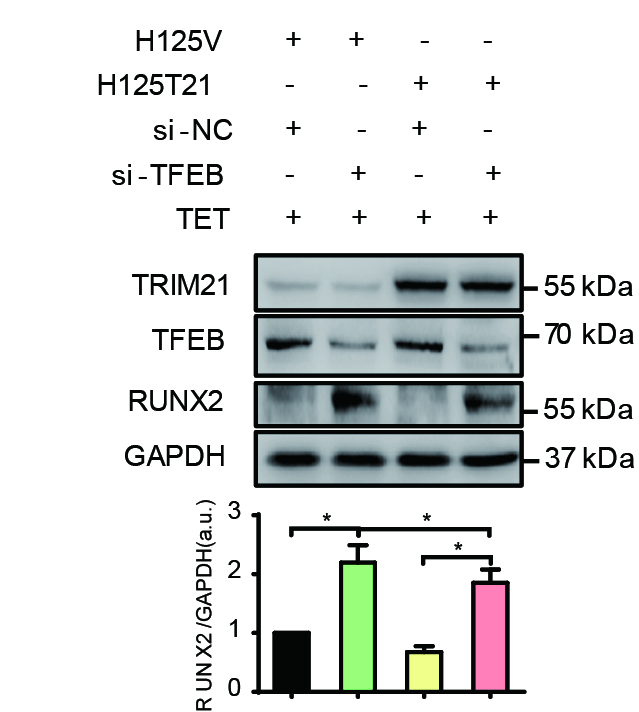

Supplement: Supplementary file 4 — Figure S3 [file 41419_2020_3364_MOESM4_ESM.tif]
