# Supplementary material for: TRIM21-regulated Annexin A2 plasma membrane trafficking facilitates osteosarcoma cell differentiation through the TFEB-mediated autophagy
Source: Cell Death Dis. 2021 Jan 6;12(1):21. doi: 10.1038/s41419-020-03364-2 (PMC7790825; doi:10.1038/s41419-020-03364-2)
Supplement: Supplementary file 5 — Supplementary Material [file 41419_2020_3364_MOESM5_ESM.docx]

**Supplementary data**

**Methods:**

**Mass spectrometry analysis of the proteins interacting with TRIM21.** The proteins interacting with TRIM21 were identified with the method previously described[^15^](#_ENREF_15). Briefly, U2-OS cells stably expressing H125-TRIM21 were used to perform co-IP experiment after TET inducting the expression of TRIM21. The immune complexes of TRIM21 were eluted from beads with 200 mmol/l NH2CH2COOH (pH 2.5) and immediately neutralized with 1 M NH4HCO3 (pH 8.8), then were subjected to in-solution digestion and liquid chromatography-tandem mass spectroscopy (LC-MS/MS) analysis. The typical parameters of mass spectrometry include a spray voltage of 2 kV, heated capillary temperature of 320°C, resolution of 60,000 at m/z 400, scan range of 350-1500 m/z, repeat duration of 30 s, and exclusion duration of 90 s.

Raw data were automatically processed by the Proteome Discoverer v2.1.1.21 (Thermo) against a database of Uniport-Humo with the default settings. 0.01 was set as the false discovery rate to determine protein and peptide. For each identified protein, at least two unique peptides were required.

**Supplementary Figure and table legends**

**Fig. S1 The effects of TRIM21 on the expression of ANXA2.** **a** U2-OS cells stably expressing H125-TRIM21 or H125-V were treated with TET (10 μg/ml) to induce the over-expression of TRIM21, or U2-OS cells were transfected with Flag-TRIM21 or Flag-vector to overexpress TRIM21. Then the cells were harvested for western blotting assay with the indicated antibodies. **b** U2-OS cells stably knocking down TRIM21 (sh-TRIM21#1 and sh-TRIM21#2), or U2-OS cells transfected with siRNA of TRIM21 were harvested for western blotting assay with the indicated antibodies. **c** U2-OS cells transfected with Flag-TRIM21 or Flag-vector were harvested for qRT-PCR assay of the expressions of TRIM21 and ANXA2 (n = 3, SEM). **d** U2-OS cells stably knocking down TRIM21 (sh-TRIM21#1 and sh-TRIM21#2) were harvested for qRT-PCR assay of the expressions of TRIM21 and ANXA2 (n = 3, SEM). ***: p<0.001; ns: no significance. All data were representative of three independent experiments.

**Fig. S2 The effects of autophagy on OS cell differentiation.** OS cells were serum deprivation for 4 h (starvation) to induce autophagy (**a**) or treated with CQ (100 μM) for 2 h to inhibit endogenous autophagy (**c**). Then the cells were harvested and performed western blotting assay with the indicated antibodies. The corresponding quantitative analyses of RUNX2/GAPDH and LC3-II/GAPDH were shown in **b** and **d** (n = 3, SEM). **e** OS cells were treated with CQ (100 μM) or in combination with CHX (10 μg/ml) for 12 h and performed western blotting assay with the indicated antibodies. **f** The quantitative analyses of RUNX2/GAPDH in **e** (n = 3, SEM). **g** OS cells were treated with CQ (100 μM) or in combination with CHX (10 μg/ml) for 12 h and performed qRT-PCR assay to analyze the mRNA expression of RUNX2 (n = 3, SEM). **h** Three types of OS cells including MG63, U2-OS and Saos-2 were harvested for western blotting assay with the indicated antibodies. **i** Three types of OS cells including MG63, U2-OS and Saos-2 were harvested to test ALP activity with ELISA assay (n = 3, SEM). *: p<0.05; **: p<0.01. All data were representative of three independent experiments.

**Fig. S3** U2-OS cells stably expressing H125-TRIM21 or H125-V were transfected with si-NC or si-TFEB and treated with TET as indicated to perform western blotting assay. RUNX2/GAPDH was shown in its lower panel (n = 3, SEM). *: p<0.05.

Table S1 **The first six proteins interacting with TRIM21 identified by LC-MS/MS**.
